# The Mosquito and Malaria

**Published:** 1903-01

**Authors:** R. Menger

**Affiliations:** San Antonio, Texas


### Correspondence.

### The Mosquito and Malaria.

San Antonio, Texas, December 19, 1902.

*Editor Texas Medical Journal*:

Your late issue, among other interesting medical literature, has an article headed with the above title by Dr. Perkins, Sabine Pass. In reading the article through I beg to protest against some of the suggestions—with all due respect to the doctor. He states that "the mosquito lights on his victim and aspirates—that is, sucks from his victim blood with which he fills himself. He injects nothing, nor can he do it. The rattlesnake inflicts a wound and injects into it a deadly poison. The mosquito has no organ by which he can inject. He can only aspirate—that is, suck the blood from his victim." This is all true and well understood. I beg to remark at the same time, though it must be remembered, that like in all other blood-drawing insects, the anatomical arrangement of the mosquito's mouth-parts are such that besides the real sucking tube they are supplied with a number of sharp dagger-like *lancets.* These delicate lancets are hidden in the cylindrical sheath of the mosquito's sucking tube, but, during the process of "biting" they are dislodged and gradually thrust into the cutis down to the capillary layer. When this sort of "scarification process" is started, the mosquito, through muscular action, sets his elastic sucking-tube into motion and aspirates the effused capillary blood. Is it then, doctor, not possible for a mosquito, or any other blood-drawing insect, to inoculate during the same process of scarification and application of the end part of the proboscis some pathogenic material, either corpuscles, bacteria or morbid tissue remnants—previously collected and adherent to the probosces or the lancets into the scarified cuticle and thence into the general circulation? We know that anthrax bacilli and other pathogenic material is thus inoculated by certain species of flies. I am well aware that we often have to deal with mere theories in some such obscure, undefinable phenomena, especially in epidemic diseases; still, I cling to the well founded probability of communication of certain obscure diseases through just some such media as the mosquito or certain fly species. I can not believe that the United States Government and other governments would have accepted the mosquito inoculation theory had they not had positive proof of same through indisputable practical experimentations in *malarious* districts where certain species of mosquitoes abound and produce malarial or yellow fever, after the subjects had exclusively been exposed to the bites of these insects. You say, doctor, the coast country of Texas is the most healthy and the most free of malarial diseases of any part of the State. "I know families who have lived in this part of the State for more than twenty years who have never had a chill nor taken a dose of quinine. How can such an extraordinary immunity from malarial diseases be accounted for if mosquitoes are the chief factors in imparting malaria to man?" Why? Doctor, I beg your pardon, but don't it seem more plausible that if your or the coast's district were full of "malaria," the liability of communication and infection through the mosquito should be simply immense? Our airied Texas inland towns and the coast, with its salt-laden Gulf breeze have not much use for the "malaria" and less so for the "malaria" mosquito—not our mean little culex communis irritans! I am rather inclined to believe (and as expressed some time ago in an illustrated article of mine in the TEXAS MEDICAL JOURNAL) that also other so-called infectious diseases (scarlatina, measles, typhoid and typhus fever and others), can be contracted through means of inoculation by the mosquito or flies. A person—children in particular—may be bitten hundreds of times by such pests, and remain in good health; if, though, this happened through a mosquito, or fly, that had lately fed on some person afflicted with such communicable diseases, why should it not be possible that such bitten person be *inoculated?* During the process of scarification of a mosquito or a fly, generally a severe pain is felt (from the insect's daggers penetrating the skin and irritating the nerve filaments) and nearly invariably and involuntarily the bitten part is "rubbed in" considerably with the hand or fingers to lessen the pain. During this process, then—a sort of massage—any particles that may have been adherent to the insect's proboscis or the daggers certainly are also liable to be absorbed through the capillaries or lymphatics with its following sequelæ.

May we not, and for such good reasons, in certain isolated instances of obscure infection, declare that either a mosquite or some fly was the prime cause of such infection? How often are we perfectly helpless to explain the real cause of a certain case of infectious disease, for instance, a case of scarlatina or typhoid fever? Lately typhoid fever cases were very numerous in San Antonio. Not alone the drinking water, at certain places, but also the milk supply, and still later the vegetables from irrigated sewer farms were blamed as a prime cause. Now, as is well known, it will happen that a typical case of typhoid fever will occur in a family where all these possible causes of infection can be absolutely stricken out. I had such a case lately. A child, about fourteen years old, was stricken down with all the symptoms of typhoid fever. The child is the fifth child of a very healthy family, never was sick before and never was exposed to any infection as far as can be ascertained. She was seriously sick for five weeks, with temperature lasting over three weeks over 104° and all inquiry as to the probable cause of her illness was absolutely negative. She fully recovered and no one else of the family was infected subsequently. Now, in this case, all had the same food (at the time the child sickened), all drank the same water and all remained well after eating the same vegetables, etc., etc. Is it not then, in this case probable that either a mosquito or a fly infected the child? At least as a "last resort" this etiology of the case perhaps comes nearer to this assumption than any other cause—who can tell?

R. MENGER, M. D.

